# Trends and factors associated with mobile phone ownership among women of reproductive age in Bangladesh

**DOI:** 10.1371/journal.pgph.0001889

**Published:** 2023-05-19

**Authors:** Gulam Muhammed Al Kibria, Jannatun Nayeem

**Affiliations:** 1 Johns Hopkins University Bloomberg School of Public Health, Baltimore, Maryland, United States of America; 2 Comilla Medical College, Comilla, Bangladesh; University of Toronto Temerty Faculty of Medicine, CANADA

## Abstract

Despite a significant increase in mobile phone ownership over the past few decades, this remains low among women in many developing countries, including Bangladesh. This cross-sectional study analyzed Bangladesh Demographic and Health Survey (BDHS) 2014 and 2017–18 data to investigate the prevalence (with 95% confidence intervals [CI]), trends, and factors associated with mobile phone ownership. We included data of 17854 and 20082 women from BDHS 2014 and BDHS 2017–18, respectively. Participants’ mean age was 30.9 (standard error [SE]: 0.09) and 31.4 (SE: 0.08) years in 2014 and 2017–18, respectively. The overall ownership was 48.1% (95% CI: 46.4%-49.9%) in 2014 and 60.1% (95% CI: 58.8%-61.4%) in 2017–18. From 2014 to 2017–18, the prevalence of mobile phone ownership increased according to most background characteristics, especially for those with lower ownership in 2014. For instance, about 25.7% (95% CI: 23.8%-27.6%) women without any formal education owned a mobile phone in 2014, the prevalence increased to 37.5% (95% CI: 35.5%-39.6%) among them in 2017–18. The following factors were associated with ownership in both surveys: age, number of children, work status, education level of women and their husbands, household wealth status, religion, and division of residence. For instance, in 2014, compared to women with no formal education, women with primary, secondary, and college/above education, respectively, had the adjusted odds ratio (AOR) of 1.8 (95% CI: 1.7–2.0), 3.2 (95% CI: 2.9–3.6), and 9.0 (95% CI: 7.4–11.0), and in 2017–18 these AORs were 1.7 (95% CI: 1.5–1.9), 2.5 (95% CI: 2.2–2.8), and 5.9 (95% CI: 5.0–7.0). The ownership of mobile phones has increased, and the socioeconomic differences in ownership have declined. However, some women groups had consistently lower ownership, especially women with low education level, low educated husbands, and low wealth status.

## Introduction

The past two decades have seen rapid developments and advancement in mobile technology, and the number of mobile phone users has grown exponentially all over the world [1,2]. In the early 2000s, the increase in ownerships, subscriptions, and use of mobile phones was slow in low- and middle-income countries (LMICs) compared to high-income countries (HICs), however, in recent years, the mobile subscription rate in many LMICs has become similar to the rate in HICs [1]. According to the International Telecommunication Union, the global mobile subscription rate was 106 per 100 people in 2020. This rate was 103 and 120 per 100 people in LMICs and HICs, respectively [1]. The use of mobile phone in LMICs also provides an opportunity to boost economic, health, educational, and technological development [3–6]. Bangladesh is also an LMIC where mobile phone subscriptions and ownership have increased rapidly in past few decades. The mobile subscription rate was 0.22 per 100 people in 2000; this rate increased to 6, 46, 84, and 107 per 100 people in 2005, 2010, 2015, and 2020, respectively. Similarly, Bangladesh Demographic and Health Survey (BDHS) 2014 found the ownership of mobile phones as 52% among men and 18% among women; BDHS 2017–18 found the proportions as 74% among men and 47% among women [7,8]. Evidence from Bangladesh also suggests that mobile phone use has contributed to socioeconomic development of the country, including health care receipt of women [9–11]. For instance, Tang et al. analyzed BDHS 2014 data and found that mobile phone use for pregnancy-related causes increases the likelihood of utilizing antenatal care and hospital delivery [12]. As Bangladesh and many other LMICs are currently dealing with high maternal and neonatal mortality along with low maternal health service utilization, having a mobile phone can positively influence the maternal and child health in these countries [10,12]. Further, mobile phone is the primary means to use internet in Bangladesh and other similar LMICs. It is the principal means to reach the underserved population in hard-to-reach areas [6,13].

Although all data show that the ownership and subscription of mobile phones have increased in the country, these remain lower among women compared to men [14,15]. Furthermore, some previous reports suggest that ownership and use are disproportionately lower among women with low socioeconomic status (i.e., women with low education level or wealth) [12,16,17], however, there has been a lack of recent investigation about this in Bangladesh. It is important to increase the ownership of mobile phones among women for not only socioeconomic development but also for empowering women and improving maternal and child health conditions [9–11]. Understanding the trend of mobile phone ownership over a period could help designing interventions to target increasing it among women who consistently have lower ownership. This study attempted to address these gaps in the literature and examined prevalence, trends, and factors associated with mobile phone ownership among women of reproductive age in Bangladesh. Findings of this study would be helpful to increase mobile phone ownership and socioeconomic development among women in Bangladesh and other similar LMICs.

## Methods

### Study design

In this cross-sectional study, we analyzed BDHS 2014 and BDHS 2017–18 data. These nationally representative surveys collected data on most major demographic and health indicators in Bangladesh. These covered urban and rural parts of all administrative divisions. Mitra and Associates, a private research firm, implemented both surveys. About two hundred data collectors were recruited and trained to conduct interviews. Data collection for BDHS 2014 took place from June to November 2014. BDHS 2017–18 data were collected between October 2017 and March 2018 [7,8].

### Sample design and coverage

In both BDHS, based on the housing and population census of 2011, a sampling frame was prepared. This frame had a list of enumeration areas (EA). Women were interviewed in households. The households were selected from the EAs in two stages. In BDHS 2014, a total of 600 EAs were selected, 393 from rural and 207 from urban regions. For BDHS 2017–18, a total of 675 EAs were selected, 425 from rural and 250 from urban regions. A higher number of EAs were selected from rural regions due to the population distribution of the country. In second stage, about 30 households were selected randomly from every EA. This produced nationally representative samples to have reliable estimates of studied demographic and health indicators. Details of both BDHS, including survey designs, methodologies, sample size estimations, questionnaires, and results, are reported and available online [7,8]. The response rate for the surveys was 98%. For the present study, we included 17,854 and 20,082 women from 17,989 and 20,160 households during BDHS 2014 and 2017–18, respectively.

### Study variables

The outcome variable of the present study was ownership of a mobile phone by a woman of reproductive age. It was based on a single question, *“Does (NAME) have a mobile phone*?*”*. Only two response options were provided: yes and no [7,8].

Based on literature review, data structure, and scientific plausibility, following study variables were selected to investigate as potential associated factors: women’s age, parity, religion, women’s work status, women’s education level, husband’s education level, household wealth status, and rural-urban place and division of residence. Women’s age was grouped as 15–24, 25–34, and 35–49 years. Parity was categorized based on the number of pregnancies: no pregnancy history, 1 pregnancy, and 2 or more pregnancies. Women reported their work status (i.e., “Are you currently working?”). The education level of women and their husbands was categorized as no formal education, primary education (i.e., 1 to 5 school years), secondary education (i.e., 6 to 10 school years), and college or above education (i.e., 11 or more school years). The household wealth status was obtained by stratifying the household wealth index score into quintiles: poorest, poorer, middle, richer, and richest; the score was obtained by principal component analyses of the basic household construction materials (e.g., floor, roof, and walls), sources of drinking water, sanitation facilities, presence of electricity, and other household belongings. BDHS 2014 had seven divisions: Dhaka, Chittagong, Rajshahi, Khulna, Barisal, Rangpur, and Sylhet [7]. In BDHS 2017–18, one more division was added, Mymensingh [8]. Women reported the religion they follow, as most people in Bangladesh are Muslims, we categorized the religion variable as Islam and others by grouping religions other than Islam as others [7,8].

### Statistical analysis

First, we described and compared demographic and socioeconomic characteristics of the study participants by survey years. We reported weighted percentages (%) and numbers (n) for categorical variables and the mean with standard errors (SE) for continuous variables. Next, we reported the prevalence (%) of mobile phone ownership by these sociodemographic characteristics in BDHS 2014 and BDHS 2017–18. The samples according to ownership were then compared using Rao Scott’s chi-square tests for both survey periods. We conducted unadjusted multilevel logistic regression to report the unadjusted odds ratio (UOR) with 95% confidence interval (CI). Variables that were significantly associated with mobile phone ownership in unadjusted analyses were adjusted into multivariable multilevel logistic regression analyses to report the adjusted OR (AOR) with 95% CI. We checked for multicollinearity using variance inflation factors. Factors associated with mobile phone ownership were reported separately for both survey years. We performed the analysis using Stata/SE 14.0 (Stata Corporation, College Station, TX, USA) [18].

## Results

A total of 17854 and 20082 women were included from BDHS 2014 and BDHS 2017–18, respectively (Table 1). The mean age of the participants was 30.9 (SE: 0.09) and 31.4 (SE: 0.08) years in 2014 and 2017–18, respectively. Overall, BDHS 2017–18 participants were more likely to be older (35-49-year-olds: 37.2% vs 34.6%), employed (47.8% vs 33.1%), formally educated (83.6% vs 75.0%), and have educated husbands (74.0% vs 68.0%) than BDHS 2014 participants. The overall ownership was 48.1% (95% CI: 46.4%-49.9%) and 60.1% (95% CI: 58.8%-61.4%) in 2014 and 2017–18, respectively. From 2014 to 2017–18, the prevalence of mobile phone ownership increased according to most sociodemographic and socioeconomic characteristics, especially among women with lower ownership in 2014. For instance, among women without any formal education, the prevalence was 25.7% (95% CI: 23.8%-27.6%) in 2014, which increased to 37.5% (95% CI: 35.5%-39.6%) in 2017–18.

**Table 1:** Study sample and mobile phone ownership among reproductive aged women, BDHS 2014 and 2017–18.

Variable	n (%)			Mobile Phone Owners, % (95% CI)		
	BDHS 2014	BDHS 2017–18	p-value	BDHS 2014	BDHS 2017–18	p-value
Overall	N = 17854	N = 20082	--	48.1 (46.4, 49.9)	60.1 (58.8, 61.4)	<0.001
Age (in Year)						
Mean (SE)	30.9 (0.09)	31.4 (0.08)	<0.001	---	---	
15–24	5228 (29.4)	5574 (27.7)	<0.001	48.0(45.7, 50.3)	59.3(57.6, 61.0)	<0.001
25–34	6396 (36.0)	7052 (35.1)		54.0(51.9, 56.2)	68.0(66.5, 69.5)	<0.001
35–49	6136 (34.6)	7494 (37.2)		42.1(40.0, 44.1)	53.3(51.7, 55.0)	<0.001
Parity						
No Children	1698 (9.6)	2019 (10.0)	0.19	51.9(48.7, 55.2)	59.4(56.7, 62.0)	0.001
1	3933 (22.1)	4288 (21.3)		53.6(51.3, 56.0)	65.7(64.0, 67.4)	<0.001
2 or More	12129 (68.3)	13813 (68.7)		45.8(44.1, 47.5)	58.5(57.2, 59.8)	<0.001
Respondent currently working						
No	11875 (66.9)	10497 (52.2)	<0.001	49.5(47.7, 51.3)	65.7(64.1, 67.2)	<0.001
Yes	5885 (33.1)	9623 (47.8)		45.4(43.2, 47.7)	54.0(52.6, 55.4)	<0.001
Women’s education level						
No Education	4433 (25.0)	3336 (16.6)	<0.001	25.7(23.8, 27.6)	37.5(35.5, 39.6)	<0.001
Primary	5181 (29.2)	6290 (31.3)		40.7(38.7, 42.7)	52.5(50.8, 54.1)	<0.001
Secondary	6642 (37.4)	7965 (39.6)		60.2(58.2, 62.1)	67.1(65.6, 68.5)	<0.001
College/Above	1505 (8.5)	2529 (12.6)		86.7(84.4, 88.8)	87.0(85.3, 88.5)	0.86
Husband’s education level						
No Education	4709 (26.5)	4090 (20.3)	<0.001	28.8(26.9, 30.8)	43.6(41.6, 45.7)	<0.001
Primary	4661 (26.2)	6095 (30.3)		42.3(40.1, 44.5)	52.4(50.7, 54.0)	<0.001
Secondary	5063 (28.5)	5687 (28.3)		55.6(53.3, 57.9)	67.2(65.6, 68.7)	<0.001
College/Above	2358 (13.3)	3100 (15.4)		81.7(79.6, 83.6)	83.9(82.3, 85.4)	0.091
Not Currently Married	970 (5.5)	1147 (5.7)		49.2(44.7, 53.7)	60.6(57.4, 63.7)	<0.001
Religion						
Muslim	16001 (90.1)	18235 (90.6)	0.71	48.9(47.2, 50.5)	60.6(59.4, 61.9)	<0.001
Other	1760 (9.9)	1884 (9.4)		41.5(35.7, 47.6)	55.3(51.1, 59.5)	<0.001
Household Wealth Status						
Poorest	3353 (18.9)	3757 (18.7)	0.96	23.7(20.6, 27.1)	39.7(37.9, 41.6)	<0.001
Poorer	3400 (19.1)	3969 (19.7)		32.6(30.2, 35.1)	47.9(45.8, 49.9)	<0.001
Middle	3550 (20.0)	4064 (20.2)		47.4(44.8, 50.0)	59.7(57.8, 61.6)	<0.001
Richer	3735 (21.0)	4165 (20.7)		55.8(53.2, 58.4)	67.2(65.3, 69.1)	<0.001
Richest	3722 (21.0)	4165 (20.7)		77.3(75.2, 79.3)	83.5(81.8, 85.1)	<0.001
Place of residence						
Urban	4977 (28.0)	5692 (28.3)	0.90	60.7(57.6, 63.6)	69.9(68.0, 71.8)	<0.001
Rural	12783 (72.0)	14427 (71.7)		43.2(41.4, 45.1)	56.3(54.8, 57.7)	<0.001
Division of residence						
Dhaka	6132 (34.5)	5064 (25.2)	0.004	52.3(49.1, 55.5)	69.0(66.1, 71.8)	<0.001
Chittagong	3285 (18.5)	3618 (18.0)		61.7(57.0, 66.1)	70.6(67.6, 73.4)	0.001
Barisal	1108 (6.2)	1134 (5.6)		52.7(48.0, 57.3)	63.9(60.7, 66.9)	<0.001
Khulna	1850 (10.4)	2348 (11.7)		41.0(37.7, 44.3)	54.9(51.8, 58.1)	<0.001
Rajshahi	2095 (11.8)	2808 (14.0)		37.6(34.0, 41.3)	50.5(47.7, 53.4)	<0.001
Rangpur	2077 (11.7)	2402 (11.9)		32.5(30.0, 35.1)	46.7(43.9, 49.5)	<0.001
Sylhet	1214 (6.8)	1193 (5.9)		41.9(36.7, 47.3)	55.5(52.2, 58.8)	<0.001
Mymensingh	--	1553 (7.7)			53.6(50.4, 56.8)	

Abbreviations: BDHS: Bangladesh Demographic and Health Survey.

Table 2 compares the characteristics of sample according to mobile phone ownership in 2014 and 2017–18. In both periods, mobile phone owners were younger (i.e., less than 35 years of age), had one child, did not work, had higher levels of education, had husbands with higher levels of education, had a higher household wealth status, and lived in urban areas. For example, in 2014 and 2017–18, among mobile phone owners, the proportions of college/above-educated women were 15.3% and 18.2%, respectively, while among those without ownership, these proportions were 2.2% and 4.1%, respectively.

**Table 2:** Comparison of study samples by mobile phone ownership, BDHS 2014 and 2017–18.

Variable	BDHS 2014			BDHS 2017–18		
	Yes	No	p-value	Yes	No	p-value
Age (in Year)						
15–24	2510 (29.4)	2718 (29.5)	<0.001	3307 (27.3)	2266 (28.3)	<0.001
25–34	3457 (40.4)	2940 (31.9)		4795 (39.6)	2257 (28.1)	
35–49	2581 (30.2)	3556 (38.6)		3995 (33.0)	3498 (43.6)	
Parity						
No Children	882 (10.3)	816 (8.9)		1199 (9.9)	820 (10.2)	
1	2110 (24.7)	1823 (19.8)	<0.001	2819 (23.3)	1469 (18.3)	<0.001
2 or More	5555 (65.0)	6574 (71.4)		8080 (66.8)	5733 (71.5)	
Respondent currently working						
No	5873 (68.7)	6002 (65.1)	0.001	6897 (57.0)	3600 (44.9)	<0.001
Yes	2674 (31.3)	3211 (34.9)		5200 (43.0)	4422 (55.1)	
Women’s education level						
No Education	1137 (13.3)	3296 (35.8)	<0.001	1252 (10.4)	2083 (26.0)	<0.001
Primary	2109 (24.7)	3072 (33.3)		3302 (27.3)	2988 (37.2)	
Secondary	3996 (46.8)	2646 (28.7)		5343 (44.2)	2622 (32.7)	
College/Above	1305 (15.3)	200 (2.2)		2200 (18.2)	329 (4.1)	
Husband’s education level						
No Education	1357 (15.9)	3352 (36.4)	<0.001	1785 (14.8)	2305 (28.7)	<0.001
Primary	1973 (23.1)	2689 (29.2)		3193 (26.4)	2902 (36.2)	
Secondary	2816 (32.9)	2247 (24.4)		3822 (31.6)	1865 (23.3)	
College/Above	1925 (22.5)	432 (4.7)		2602 (21.5)	499 (6.2)	
Not Currently Married	477 (5.6)	493 (5.3)		695 (5.7)	452 (5.6)	
Religion						
Muslim	7817 (91.5)	8184 (88.8)	0.022	11055 (91.4)	7180 (89.5)	0.017
Other	731 (8.5)	1029 (11.2)		1042 (8.6)	842 (10.5)	
Household Wealth Status						
Poorest	795 (9.3)	2558 (27.8)	<0.001	1492 (12.3)	2264 (28.2)	<0.001
Poorer	1108 (13.0)	2292 (24.9)		1899 (15.7)	2070 (25.8)	
Middle	1682 (19.7)	1868 (20.3)		2428 (20.1)	1636 (20.4)	
Richer	2085 (24.4)	1651 (17.9)		2799 (23.1)	1366 (17.0)	
Richest	2877 (33.7)	845 (9.2)		3479 (28.8)	686 (8.6)	
Place of residence						
Urban	3019 (35.3)	1958 (21.3)	<0.001	3981 (32.9)	1711 (21.3)	<0.001
Rural	5528 (64.7)	7255 (78.7)		8116 (67.1)	6311 (78.7)	
Division of residence						
Dhaka	3208 (37.5)	2925 (31.7)	<0.001	3495 (28.9)	1569 (19.6)	<0.001
Chittagong	2027 (23.7)	1259 (13.7)		2554 (21.1)	1064 (13.3)	
Barisal	584 (6.8)	524 (5.7)		724 (6.0)	410 (5.1)	
Khulna	758 (8.9)	1092 (11.8)		1290 (10.7)	1058 (13.2)	
Rajshahi	788 (9.2)	1307 (14.2)		1418 (11.7)	1390 (17.3)	
Rangpur	674 (7.9)	1402 (15.2)		1121 (9.3)	1281 (16.0)	
Sylhet	509 (6.0)	705 (7.6)		663 (5.5)	530 (6.6)	
Mymensingh				833 (6.9)	720 (9.0)	

Abbreviations: BDHS: Bangladesh Demographic and Health Survey.

The factors that were associated with mobile phone ownership were similar in both surveys (Table 3). Overall, socioeconomic status (i.e., education level and wealth status) had dose-response relationships with mobile phone ownership in both periods. For instance, in 2014, compared to women with no formal education, women with primary, secondary, and college/above, respectively, had the AOR of 1.8 (95% CI: 1.7–2.0), 3.2 (95% CI: 2.9–3.6), and 9.0 (95% CI: 7.4–11.0), and in 2017–18 these AORs were 1.7 (95% CI: 1.5–1.9), 2.5 (95% CI: 2.2–2.8), and 5.9 (95% CI: 5.0–7.0). Women with 25–34 years of age had the highest odds in both years: 1.7 (95% CI: 1.5–1.8) in 2014 and 1.7 (95% CI: 1.5–1.9) in 2017–18. Women with secondary- and college-educated husbands were more likely to own mobile phones too. Compared to Muslims, non-Muslims had lower adjusted odds, 0.7 (95% CI: 0.6–0.8) and 0.8 (95% CI: 0.7–0.9) in 2014 and 2017–18, respectively. Division of residence also had an association with the outcome variable.

**Table 3:** Factors associated with mobile phone ownership, BDHS 2014 & 2017–18.

	BDHS 2014		BDHS 2017–18	
Variables	COR	AOR	COR	AOR
Age (in Year) (Ref: 15-24-year-olds)				
25–34	1.5*** (1.3,1.6)	1.7*** (1.5,1.8)	1.5*** (1.4,1.6)	1.7*** (1.5,1.9)
35–49	0.9** (0.8,1.0)	1.2*** (1.1,1.4)	0.8*** (0.8,0.9)	1.2** (1.0,1.3)
Parity (Ref: 2 or More)				
No Children	1.2** (1.1,1.3)	0.8*** (0.7,0.9)	1.0 (0.9,1.1)	0.7*** (0.6,0.8)
1	1.4*** (1.3,1.5)	0.9 (0.9,1.0)	1.4*** (1.3,1.5)	1.0 (0.9,1.1)
Work Status (Ref: No)				
Yes	0.9** (0.9,1.0)	1.2*** (1.1,1.3)	0.7*** (0.7,0.8)	1.0 (0.9,1.0)
Education Level (Ref: No Education)				
Primary	2.0*** (1.8,2.2)	1.8*** (1.7,2.0)	1.9*** (1.7,2.0)	1.7*** (1.5,1.9)
Secondary	4.6*** (4.2,5.0)	3.2*** (2.9,3.6)	3.5*** (3.2,3.8)	2.5*** (2.2,2.8)
College/Above	24.1*** (20.4,28.4)	9.0*** (7.4,11.0)	13.2*** (11.5,15.1)	5.9*** (5.0,7.0)
Husband’s Education Level (Ref: No Education)				
Primary	1.7*** (1.5,1.8)	1.1 (1.0,1.2)	1.3*** (1.2,1.5)	1.0 (0.9,1.1)
Secondary	3.1*** (2.8,3.3)	1.2*** (1.1,1.4)	2.5*** (2.3,2.7)	1.3*** (1.2,1.4)
College/Above	11.6*** (10.3,13.1)	2.1*** (1.8,2.4)	7.4*** (6.6,8.4)	1.8*** (1.6,2.1)
Not Currently Married	2.2*** (1.9,2.6)	1.7*** (1.4,2.0)	1.9*** (1.6,2.1)	1.5*** (1.3,1.7)
Religion (Ref: Muslim)				
Other	0.8*** (0.7,0.9)	0.7*** (0.6,0.8)	0.9** (0.8,1.0)	0.8*** (0.7,0.9)
Household Wealth Status (Ref: Poorest)				
Poorer	1.7*** (1.5,1.9)	1.5*** (1.3,1.7)	1.4*** (1.3,1.5)	1.3*** (1.1,1.4)
Middle	2.9*** (2.6,3.2)	2.0*** (1.8,2.3)	2.2*** (2.0,2.4)	1.7*** (1.5,1.8)
Richer	4.7*** (4.2,5.2)	2.8*** (2.5,3.2)	3.0*** (2.7,3.3)	2.0*** (1.8,2.2)
Richest	13.0*** (11.5,14.6)	5.2*** (4.5,6.0)	7.7*** (7.0,8.6)	3.6*** (3.2,4.1)
Place of Residence (Ref: Urban)				
Urban	Ref. (1.0)	Ref. (1.0)	Ref. (1.0)	Ref. (1.0)
Rural	0.5*** (0.5,0.5)	0.9 (0.9,1.0)	0.6*** (0.6,0.6)	1.0 (0.9,1.1)
Division of Residence (Ref: Dhaka)				
Chittagong	1.4*** (1.2,1.5)	1.6*** (1.4,1.8)	1.0 (0.9,1.2)	1.2* (1.0,1.3)
Barisal	1.0 (0.9,1.1)	1.4*** (1.2,1.6)	0.8*** (0.7,0.9)	1.1 (1.0,1.2)
Khulna	0.6*** (0.6,0.7)	0.7*** (0.6,0.8)	0.6*** (0.5,0.7)	0.6*** (0.6,0.7)
Rajshahi	0.6*** (0.5,0.6)	0.6*** (0.6,0.7)	0.5*** (0.4,0.5)	0.6*** (0.5,0.7)
Rangpur	0.5*** (0.4,0.5)	0.7*** (0.6,0.8)	0.4*** (0.4,0.5)	0.6*** (0.5,0.7)
Sylhet	0.7*** (0.6,0.7)	1.0 (0.9,1.2)	0.6*** (0.5,0.7)	0.8*** (0.7,0.9)
Mymensingh	Not applicable	Not applicable	0.5*** (0.5,0.6)	0.8*** (0.7,0.9)

Abbreviations: BDHS: Bangladesh Demographic and Health Survey, COR: Crude odds ratio, AOR: Adjusted odds ratio.

## Discussion

This study investigated the prevalence, trends, and factors associated with mobile phone ownership among women of reproductive age in Bangladesh. We found that women with higher socioeconomic status were more likely to own mobile phones in the country. We also observed that ownership increased across most sociodemographic and socioeconomic groups between 2014 and 2017–18. This study adds to the growing body of literature investigating the disparities in mobile phone ownership or the digital divide of mobile phone ownership in LMICs such as Bangladesh.

One welcome finding of the present study is the increase in mobile phone ownership observed between 2014 and 2017–18, which was in line with the country’s socioeconomic development (e.g., education level and wealth status). We also noted a higher proportion of women with higher education levels or higher educated husbands in BDHS 2017–18 compared to BDHS 2014, which has been a consistent trend since BDHS 1993–94 [7,8,19]. Although BDHS did not collect personal income information, these two variables are known to be significantly associated with income. The average income of Bangladeshi people has steadily increased during this period [7,8,19].

We also noticed that the socioeconomic differences in mobile phone ownership have reduced in BDHS 2017–18 compared to BDHS 2014. In other words, the socioeconomic differences in adjusted odds of owning a mobile phone were lower in 2017–18 compared to 2014 [7,8]. It indicates that the impacts of characteristics (e.g., socioeconomic status) that differentiate mobile phone owners from non-owners have also declined [17]. In the early period, high mobile phone costs, the lack of willingness to bear the cost, and slow adoption of this new technology resulted in a gap in ownership, but over time, the impact has reduced [17,20]. The increase in ownership did not change substantially among women with a college education or women with college-educated husbands, indicating that the ownership has reached a plateau stage among these population groups. Sociocultural norms in Bangladesh may also prevent some women from owning mobile phones. In the patriarchal system where men are the primary controllers of finances and all family decisions, women may not require or have permission to own mobile phones [17,21–23].

Many studies are conducted in Bangladesh and other similar LMICs using mobile phone surveys, those surveys cannot be generalizable without increasing ownership among all socioeconomic groups [24–27]. The socioeconomic disparities in mobile phone ownership among Bangladeshi women indicate that future studies should explore implementing programs and policies to reduce this digital divide. The government of Bangladesh currently works in partnership with multiple local and national non-governmental organizations to empower women [7,8]. They can also work with them to make mobile phone ownership easier, especially providing low-cost mobile phone and internet. They can also work with mobile phone companies to increase access or ownership. Considering the advantages of mobile phone ownership or use, minimizing the gap will also help minimize socioeconomic disparities in health or economic outcomes [9–11].

Overall, more than one-third of the women did not own any mobile phone. We did not ask them if any other family member (e.g., husband or children) owned a mobile phone. However, it is also important to ensure mHealth or other coverage for this group of women to take full advantage of mobile phone technology [9–11]. Future studies should also investigate ways to reach them.

This study has several notable strengths. We reported the prevalence, trends, and factors using two datasets from Bangladesh, covering rural and urban regions of all administrative divisions has made the study nationally representative and generalizable to reproductive-aged women of the country. The surveys had large sample sizes that enabled us to perform subgroup analyses, and the response rates were high. The survey used standardized validated questionnaires that increased the authenticity of our findings.

The limitations of the present study also merit discussion. We did not consider some variables (e.g., network coverage in the area) that may impact the ownership. We did not account for whether a woman did not want to use a phone or if her family did not allow her to own it. This was a cross-sectional study, which limits our understanding of causal associations due to the lack of the evidence about the temporal relationship between explanatory variables and outcomes.

## Conclusion

The present study found that the ownership of mobile phones has increased significantly among women of reproductive age in Bangladesh and the socioeconomic differences in ownership have declined. However, some women groups had consistently lower ownership, to increase its overall level and increase the advantage of mobile phone technology, it is important to increase the coverage among them, especially among women with lower education levels, lower educated husbands, and wealth status.
